# An Evaluation of IL-10 Encoded by Cytomegalovirus in the Prediction of Coronary Artery Disease in People Living with HIV

**DOI:** 10.3390/pathogens15020192

**Published:** 2026-02-09

**Authors:** Shelley Waters, Luna-Faye Veld, Silvia Lee, Anna C. Hearps, Janine Trevillyan, Jennifer F. Hoy, Patricia Price

**Affiliations:** 1 Curtin Medical Research Institute, Curtin University, Bentley 6102, Australia; shelley.waters@uwa.edu.au (S.W.);; 2 Medical School, University of Western Australia, Nedlands 6009, Australia; silvia.lee@uwa.edu.au; 3 Harry Perkins Institute of Medical Research, Nedlands 6009, Australia; 4 Pathwest Laboratory Medicine, Department of Microbiology and Infectious Diseases, Murdoch 6150, Australia; 5 Life Sciences Discipline, Burnet Institute, Melbourne 3004, Australia; 6 Department of Infectious Diseases, Monash University, Melbourne 3004, Australia; 7 Department of Infectious Diseases, Peter Doherty Institute of Infection and Immunity, University of Melbourne, Melbourne 3004, Australia; 8 Department of Infectious Diseases, Austin Health, Melbourne 3084, Australia; 9 Department of Infectious Diseases, Alfred Hospital, Melbourne 3004, Australia

**Keywords:** HIV, cytomegalovirus, coronary artery disease, interleukin 10, cmvIL-10

## Abstract

Cytomegalovirus (CMV) seropositivity associates with cardiovascular disease in healthy adults, but associations are unclear in people living with HIV (PLWH) despite their high CMV burden. However, CMV antibody levels correlated with inflammatory biomarkers only in PLWH who subsequently developed coronary artery disease (CAD), so the effects of CMV in an individual may vary. Here we investigate the role of CMV-encoded interleukin-10 (cmvIL-10) in PLWH on anti-retroviral therapy. Plasma levels of cmvIL-10 and antibodies reactive with a cmvIL-10 peptide or a lysate of CMV-infected fibroblasts were assessed in PLWH with or without CAD. cmvIL-10 was assessed at diagnosis/selection (T0) and 12 months earlier (T-12), with anti-cmvIL-10 also assessed at −24 and −36 months (n = 36–58/group). Plasma cmvIL-10 was recorded as positive in 5–10 PLWH per group, irrespective of CAD status. Of 21 PLWH with detectable cmvIL-10, only six were positive at both timepoints. Anti-cmvIL-10 was measurable in all samples, at levels independent of cmvIL-10, CAD or time of sampling. Amongst PLWH without CAD, the detection of cmvIL-10 associated with higher levels of CXCL10 (T0 and T-12) and lower levels of the IL-1 receptor antagonist (IL-1Ra; T0 only). At T-12, anti-cmvIL-10 correlated with IL-1Ra in PLWH without CAD (*p* = 0.01), and sCD14 in PLWH with CAD (*p* = 0.01). Anti-cmvIL-10 correlated with VCAM-1 at several timepoints in both groups. Hence, cmvIL-10 may be produced episodically, inducing anti-cmvIL-10 peptide antibody, which may represent levels of the cytokine averaged over time. Plasma levels of cmvIL-10 and anti-cmvIL-10 antibody associated differently with inflammatory biomarkers in PLWH with and without CAD, suggesting mechanisms by which host responses to CMV may have different clinical consequences.

## 1. Introduction

Persistent infections with human cytomegalovirus (CMV) are common world-wide. Seropositivity rates are around 70% in the general population and over 90% in people living with HIV (PLWH). The virus establishes latency following primary infection, with periodic reactivations throughout life [1]. Infections in healthy adults are typically asymptomatic, but people who are immunocompromised may experience more reactivation events and end-organ disease [2].

In the general population, CMV infection (assessed by CMV-reactive IgG) increases the relative risk of developing cardiovascular disease (CVD) by 22% [3]. Accordingly, in a meta-analysis of abdominal organ transplant recipients, CMV infection or disease was associated with an increased risk of cardiovascular events [4]. People living with HIV (PLWH) are almost universally seropositive for CMV, but links between CMV and CVD are unclear. This probably reflects the paucity of CMV-negative individuals and the steady increase in antibody titres seen as PLWH first respond to anti-retroviral therapy (ART) [5].

We recently published a study of PLWH receiving ART who experienced overt coronary artery disease (CAD). They were matched by age and sex with individuals without CAD and assessed immediately prior to diagnosis (or selection) and up to 36 months earlier. Whilst levels of antibodies reactive with a lysate of CMV-infected fibroblasts were similar in PLWH with and without CAD, antibody levels correlated directly with plasma soluble (s) CD14, lipopolysaccharide binding protein (LBP), CXC10 and/or interleukin (IL)-6 12–36 months before a diagnosis of CAD. These findings were independent of HIV viraemia, defined as the presence of >200 copies/mL HIV RNA [6].

Here we seek reasons why CMV may differentially affect particular PLWH so as to promote CAD. CMV encodes several immune modulatory genes that have potential to shape both the immunological and clinical outcomes of infection. This includes *UL111a,* which encodes a viral homologue of human (h) IL-10, denoted cmvIL-10 [7,8]. cmvIL-10 signals through the native IL-10 receptor (IL-10R) with comparable affinity to hIL-10 [9]. Ex vivo cell-culture models outline a capacity for cmvIL-10 to suppress the activation and functional capacity of dendritic cells [10,11]. Furthermore, cmvIL-10 can provide positive feedback, enhancing hIL-10 secretion in vivo [12]. However, some mechanisms invoked by cmvIL-10 are distinct from those of hIL-10. This includes enhancement of CXCR4 signalling triggered by CXCL12. cmvIL-10 may enhance IL-10R–CXCR4 crosstalk via shared signalling intermediates, as both receptors activate pathways involving calcium flux, cAMP, and kinases such as PI3K and MAPK [13].

Preliminary evidence that cmvIL-10 impacts the biological consequences of persistent asymptomatic CMV infections includes our detection of cmvIL-10 in the plasma of some healthy adults who remain CMV-seronegative [14], suggesting an ability to suppress seroconversion. Moreover, healthy adults with detectable cmvIL-10 had reduced plasma levels of an inflammatory molecule associated with atherosclerosis, vascular-cell-adhesion molecule-1 (VCAM-1), and reduced proportions of terminally differentiated T-cells, a hallmark of CMV infection. In the same study, these changes were not apparent in renal transplant recipients (RTR) but detection of cmvIL-10 appeared to mark CMV burden as it associated with increased numbers of T-cells responding to CMV [15]. This may be a feature of immunocompromised individuals.

The aim of this study was to assess the expression of cmvIL-10 and its role in the development of CAD in PLWH on ART. To achieve this, we investigated relationships between the detection of cmvIL-10 and CAD events in PLWH receiving ART. Almost all were CMV-seropositive. We also evaluated antibodies reactive with a peptide specific to cmvIL-10 as a metric of levels of cmvIL-10 integrated over time.

## 2. Materials and Methods

### 2.1. Patient Cohort

The Alfred Acute Myocardial Infarction (AMI) Study in HIV (AASH) is a retrospective single-centre case-control study of CAD in PLWH treated at Alfred Hospital, Melbourne, Australia, identified from the Victorian HIV database which held records of 5721 PLWH (1996–2018) [16]. Cases had a diagnosis of CAD defined as either acute myocardial infarction, documented ischemic heart disease, coronary angiogram results consistent with moderate-to-severe CAD, or a coronary artery bypass graft procedure. These were matched 1:1 by age (^+^/_−_ 5 years) and sex with PLWH without CAD who attended the clinic within the same year as their matched case. For this substudy, archived plasma samples collected 0–1 month, 12, 24 and/or 36 months prior to the diagnosis of CAD in the matched case and control were utilised from the Victorian HIV Blood and Tissue Storage Bank (Alfred Hospital, Melbourne, Australia). Samples were available from at least 2 timepoints in all case-control pairs. We restricted our analyses to PLWH who had ≥12 months on ART at the timepoints used, because levels of CMV-reactive antibody rise during ART [5]. Standard demographics, and metrics of HIV disease and the use of abacavir were collected from the Alfred Hospital HIV database and a manual review of medical records. Current CD4 T-cell counts and risk factors for CAD were reported at T0 (immediately prior to diagnosis/selection). The AASH study was approved by the Alfred Hospital Ethics Committee (AH #205-09).

### 2.2. Inflammatory and Cardiovascular Markers

Soluble (s) CD14, CXCL10, IL-1Ra, VCAM-1, IL-6 and LBP were measured in archived plasma using customised magnetic Luminex assays (R&D Systems, Minneapolis, MN, USA). IL-6 was measured by high-sensitivity Quantikine ELISA (R&D Systems).

### 2.3. Plasma cmvIL-10

Ninety-six-well ELISA plates (half volume) were coated overnight with 50 μL goat polyclonal anti-cmvIL-10 antibody (2 μg/mL; AF117, R&D Systems) at 4 °C, washed and blocked with 2% bovine serum albumin (BSA)/phosphate-buffered saline (PBS) for 2 h. A standard curve was generated using recombinant cmvIL-10 with the BSA carrier protein (117-VL-025, R&D Systems) serially diluted in 1% BSA/PBS. Plasma samples were diluted with 1% BSA/PBS and 50 μL aliquots were transferred to the plates (2 h, room temperature). Samples were screened at 1:10 and re-run at 1:8 and 1:16 if optical densities (ODs) were equal to or higher than any blanks on the screening plate. A plasma sample containing mid-range levels of cmvIL-10 was included on each plate as an internal control. Binding was detected using 0.2 μg/mL biotinylated polyclonal goat anti-cmvIL-10 antibody (BAF117, R&D Systems) for 2 h. After further washes, 1:2000 streptavidin-conjugated HRP (BD Biosciences, Franklin Lakes, NJ, USA) diluted in 1% BSA/PBS was added for 30 min. Plates were washed and 3,3′-5,5′ tetramethylbenzidine (TMB) substrate (Sigma–Aldrich, St. Louis, MO, USA) was added, reactions were stopped with 1M H_2_SO_4_ and read at 450 nm. Samples generating ODs above all blanks on the respective plate were deemed to be positive. Any sample with an OD below all blanks were deemed negative. Samples with ODs (recorded at 1:8 or 1:10) between the highest and lowest blank on the plate are described as “trace”.

### 2.4. CMV-Reactive Antibodies

Plasma antibodies reactive with multiple viral proteins were quantitated using a lysate of fibroblasts infected with CMV strain AD169, as described previously [5,6], and are recorded in arbitrary units (AU). The inter-assay coefficient of variance was 13.9%.

**Figure 1:**
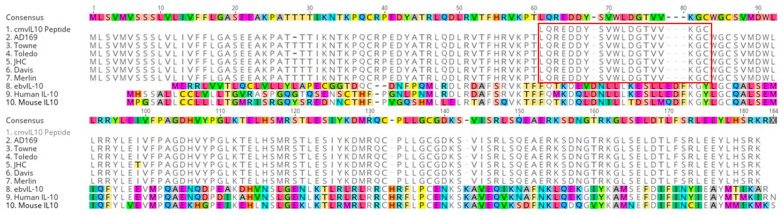
Sequence alignments of IL-10 encoded by laboratory CMV strains (2–7), Epstein–Barr Virus (8. EBV), humans (9) and mice (10). The selected cmvIL-10 peptide is included (1) and the perfect match with all CMV strains analysed is highlighted by a red box. Colours denote amino acid variations from the consensus shown in lines 2–7. The alignment was performed using Geneious alignment tool with default parameters. The image was generated using Geneious version 2025.2 created by Biomatters, Auckland, New Zealand <https://www.geneious.com>. Protein sequences were sought from NCBI. Accession numbers are in Supplementary Table S1A.

To quantitate antibodies reactive with cmvIL-10, a 19-amino-acid peptide derived from the full-length cmvIL-10 protein (LQREDDYSVWLDGTVVKGC) was synthesised by Mimotopes Pty Ltd. (Victoria, Australia). The peptide sequence was chosen with consideration to receptor binding and the dimer structure of the full-length protein, with charged amino acid side chains to optimise immunogenicity. Its specificity to cmvIL-10 is illustrated in Figure 1. The accession numbers used to derive the figure are provided (Supplementary Table S1A). We also show the result of a BLAST (version 2.17.0) search seeking random matches to our peptide. The weak matches obtained with genomes other than CMV (E-values > 1; Supplementary Table S1B) were all from environmental organisms—many from the ocean. Several are putative sequences.

Half-well plates were coated with 10 μg/mL cmvIL-10 peptide diluted in carbonate/bicarbonate buffer (overnight, 4 °C), washed and blocked with 5% BSA/PBS. Purified intravenous anti-CMV immunoglobulin (LifeBlood Australia, CSL, Melbourne, Australia) was diluted as a standard (assigned value 15.6–1000 AU). Samples, an internal positive control and standards (all in 2% BSA/PBS) were added for 2 h at room temperature, followed by washing. Goat Fc-specific anti-human IgG conjugated to peroxidase (Sigma–Aldrich) diluted 1:4000 in 2% BSA/PBS was then added for one hour. After 5 washes, TMB substrate was added and reactions were stopped with 1M H_2_SO_4_.

### 2.5. Statistical Analyses

Continuous variables were compared using non-parametric Mann–Whitney tests and presented as median (range). Categorical variables were assessed using Fisher’s Exact tests. Correlations were assessed using Spearman’s non-parametric correlation coefficients. Statistical analyses were completed using GraphPad Prism (GraphPad Software version 5.04, Boston, MA, USA). Statistical significance was defined as *p* < 0.05, but comparisons achieving 0.05 < *p* < 0.1 are noted.

## 3. Results

### 3.1. Levels of CMV-Reactive Antibody Did Not Align with a Diagnosis of CAD, but Correlated with Levels of Several Inflammatory/Cardiovascular Biomarkers in PLWH with CAD

Data for all timepoints are reported elsewhere [6], but those obtained 12 months before diagnosis/recruitment are provided here as a Supplement as they form the basis for the present study. PLWH with and without CAD had similar levels of antibody reactive with CMV lysates at all timepoints. Levels of inflammatory and cardiovascular biomarkers were also similar, with the exception of IL-6, which was present at slightly higher levels in PLWH who developed CAD. Exposure to abacavir aligned with CAD at T-12 months (Supplementary Table S2A) and other timepoints [6]. When PLWH with and without CAD were analysed separately, plasma levels of biomarkers (sCD14, LBP, CXCL10 and IL-6) correlated with levels of CMV-reactive antibodies only in PLWH who developed CAD (Supplementary Table S2B). These findings were independent of the presence of >200 copies/mL HIV RNA [6]. Here we investigate the role of cmvIL-10 in the observed associations between CMV metrics, biomarkers and CAD.

### 3.2. Plasma cmvIL-10 Was Measurable in a Minority of PLWH but Was Usually Not Maintained over 12 Months—All Samples Contained Measurable cmvIL-10 Antibodies

Plasma cmvIL-10 was assessed immediately prior to diagnosis/selection and 12 months earlier. Twenty-one PLWH had measurable cmvIL-10 at some time. Of these, samples from six individuals were positive at both timepoints. The incidence of positivity was independent of CAD (Table 1) and did not change over time in either group (*p* = 0.38 in PLWH with CAD). The inclusion of samples with trace levels of cmvIL-10 yielded similar trends, as only 13/34 individuals ascribed positive or trace cmvIL-10 were in the same category at both T0 and T-12.

Antibodies reactive with our cmvIL-10 peptide (as per Figure 1; Supplementary Table S1A,B) were quantifiable in all individuals, and group-wise associations found no associations with CAD 0–36 months before diagnosis/recruitment (Table 1).

### 3.3. Plasma cmvIL-10 Associated Positively with High CXCL10 and Low IL-1Ra, but Not with Antibodies Reactive with cmvIL-10 or a Lysate of CMV-Infected Cells

For these analyses, samples with traces of cmvIL-10 were excluded to create two distinct populations either positive or negative for cmvIL-10. Levels of antibody reactive with a lysate of CMV-infected fibroblasts or with our cmvIL-10 peptide were similar in samples that were positive or negative with respect to cmvIL-10 (Table 2). Hence, the detection of cmvIL-10 does not determine levels of antibody reactive with a cmvIL-10 peptide measured in the same sample.

Plasma levels of CXCL10 were significantly higher in controls (no CAD) who expressed cmvIL-10 at T0 (*p* = 0.01) and T-12 (*p* = 0.01). These values were slightly higher than were seen in PLWH with CAD at both timepoints but the numbers were small, so the difference was not significant. Amongst PLWH without CAD, those with cmvIL-10 had lower levels of IL-1Ra, at T0 (*p* = 0.003). This was not evident at T-12 or in those with CAD (Table 2).

### 3.4. Levels of Antibody Reactive with cmvIL-10 Correlated Directly with sCD14 in PLWH with CAD and with IL-1Ra in PLWH Without CAD

Levels of antibody reactive with cmvIL-10 peptide correlated variably with antibodies reactive with a lysate of CMV-infected cells. This reached significance in PLWH with CAD (Table 3; *p* = 0.01–0.03). Moreover, levels of sCD14 (an inflammatory biomarker) correlated with cmvIL-10 antibodies in PLWH who developed CAD. This was significant 12 months before CAD diagnosis (*p* = 0.01), but a trend was evident at −24 months. It mirrors the selective associations seen with antibodies reactive with the lysate of CMV-infected fibroblasts (Supplementary Table S2B).

In contrast, cmvIL-10 antibody and plasma IL-1Ra levels correlated directly only in PLWH without CAD at T-12 (*p* = 0.01; Table 3).

Levels of VCAM-1 (an endothelial activation marker) correlated with anti-cmvIL-10 12–36 months before diagnosis/selection but the level of significance varied (*p* = 0.005–0.07; Table 3). This is consistent with the marginally higher levels of VCAM-1 seen in PLWH expressing cmvIL-10 (*p* = 0.10; Table 2).

## 4. Discussion

To understand links between CMV and CAD, applicable metrics are needed to define the amount of CMV in the body and determine whether it is actively replicating. Levels of antibody reactive with CMV lysates or individual proteins have been used in most publications to date, as CMV DNA cannot be detected in most people. This creates results that must be evaluated with care. For example, we showed that the detection of CMV DNA in saliva was a significant predictor of poor vascular health as measured by resting dilation of the brachial artery (flow mediated dilation; FMD) in renal transplant recipients (RTR). However, antibodies to CMV glycoprotein-B (gB) were protective against endothelial dysfunction, as assessed by FMD [17]. In a parallel cohort, the detection of cmvIL-10 associated with seropositivity only in RTR, as three seronegative healthy adults had detectable plasma cmvIL-10. RTR with detectable cmvIL-10 had elevated interferon-γ T-cell responses to CMV antigens, whilst cmvIL-10 in healthy adults associated with reduced populations of terminally differentiated T-cells [15]. Hence, the high burdens of CMV seen in individuals with compromised immune systems may alter the pathogenic effects of the virus, including effects initiated by cmvIL-10. This is in addition to the possibility that all CMV isolates are not the same—indeed we demonstrated polymorphism in the gene encoding cmvIL-10 (*UL111a*) in clinical isolates [8].

Here we present the first study to address the expression of cmvIL-10 in plasma samples from PLWH receiving ART, and align this with a subsequent diagnosis of CAD. In the same cohort, we demonstrated that levels of CMV-reactive antibody did not align with the occurrence of CAD, but correlated significantly with inflammatory and cardiovascular biomarkers only in PLWH who developed CAD [6]. Here we show that only a small proportion of PLWH expressed cmvIL-10 in plasma, where most did not retain expression over the period studied (12 months). cmvIL-10 positivity did not align with CAD.

However, these data have a number of caveats. Firstly, the small numbers of positive samples affect the statistical power of the study. Future studies should address larger cohorts. Second, the only antibody available commercially to detect cmvIL-10 is polyclonal (raised in goats) and is used un-biotinylated for coating the ELISA plates and biotinylated for detection. Hence, with an abundance of caution, we scored samples as negative, trace and positive. The inclusion of samples with trace cmvIL-10 did not create associations with CAD (Table 1). However, their omission allowed us to confidently compare samples that were positive and negative for cmvIL-10 (Table 2), showing differences with CXCL10 and IL-1Ra.

The sensitivity of any means of detecting cmvIL-10 is problematic as the question of “how much matters?” remains open. Amongst RTR, cmvIL-10 positivity aligned with CMV-reactive T-cells (detected by interferon-γ ELISpot), but not with antibodies reactive with CMV immediate-early protein 1, gB or lysate, or with circulating CMV DNA [15]. The latter suggests that our ELISA may detect the “latency-associated” truncated isoform of cmvIL-10 (LAcmvIL-10), which can be released without active viral replication. LAcmvIL-10 does not bind IL-10R1 but can down-regulate MHC class II expression and induce the human leukocyte chemoattractant CCL8 [18].

The observed fluctuations in cmvIL-10 detection over time are consistent with the notion that all participants may express cmvIL-10 episodically. This consideration supports our decision to consider cmvIL-10 as a categorical rather than continuous variable. It also prompted the development of an anti-peptide antibody assay. The universal positivity for cmvIL-10 antibody suggests the antigen is ubiquitous. To elucidate the dynamics of cmvIL-10, ELISAs detecting the cytokine and the antibody will need to be more sensitive and to distinguish full-length from LAcmvIL-10. The peptide used here is present in both forms.

With these caveats in mind, the associations observed between cmvIL-10 antibodies, CMV-lysate antibodies and inflammatory biomarkers warrant cautious consideration. Associations between the two antibodies were moderate and variable. They may be stronger in PLWH diagnosed with CAD, but the distinction is unclear. Associations with biomarkers are discussed individually below.

1. **CXCL10:** Amongst PLWH without CAD, CXCL10 levels were higher in participants with plasma cmvIL-10 at T0 and T-12. The finding suggests CXCL10 may be induced by cmvIL-10. Taken in isolation, this association suggests CXCL10 may reduce atherogenesis in this setting. This contrasts with the traditional view of this chemokine [19], so the associations should be considered with caution. CXCL10 correlated directly with antibodies reactive with lysate from CMV-infected cells but not cmvIL-10 in PLWH with CAD at T-12 (Supplementary Table S2B; Table 3), so cmvIL-10 appears unlikely to explain all effects of CMV on CXCL10.
2. **sCD14:** Plasma levels were not affected by cmvIL-10 positivity (Table 2). However, sCD14 levels were slightly higher in PLWH with CAD at T-24 only [6] and correlated with cmvIL-10 antibodies at T-12 (and marginally at T-24) in PLWH with CAD (Table 3). Importantly, levels of sCD14 and CMV-lysate antibodies also correlated in PLWH with CAD (Supplementary Table S2B) [15], so cmvIL-10 may not explain all associations between CMV and sCD14.
3. **VCAM-1:** Weak positive correlations with cmvIL-10 antibody were evident 12–36 months before diagnosis/selection (Table 3). This was not apparent when we assessed correlations with CMV-lysate antibodies (Supplementary Table S2B) [6]. Accordingly, VCAM-1 levels were marginally higher in PLWH expressing cmvIL-10 at T-12, irrespective of CAD (Table 2). Levels of VCAM-1 did not align with CAD per se [6,16], but modulation of VCAM-1 by cmvIL-10 is plausible from our data. Plasma cmvIL-10 associated with lower VCAM-1 levels in healthy adults, but not in RTR [15]. The latter accords with present findings in PLWH.
4. **IL-1Ra:** Levels were significantly lower in PLWH without CAD expressing cmvIL-10 at T0 (Table 2). This suggests that cmvIL-10 blocks the induction of IL-1Ra in a manner that may be protective against CAD. However, cmvIL-10 antibody levels correlated directly with IL-1Ra in PLWH without CAD at T0 and T-12 (Table 3). This opens the possibility that the anti-peptide antibody may neutralise cmvIL-10 or mark the presence of other antibodies that are neutralising. Associations with IL-1Ra were not apparent with CMV lysate antibodies [6], so the findings described herein may be a direct effect of cmvIL-10. Further studies of these pathways are warranted as IL-1 is a component of the inflammasome and is implicated in CAD. Indeed IL-1Ra is increasingly prescribed as a therapeutic agent to treat manifestations of CVD [20], so links with cmvIL-10 may be important.

In conclusion, we have proposed novel pathways involving cmvL-10 by which CMV may modulate CAD in individuals with a high burden of CMV. We have shown that simple comparisons of the incidence of CMV seropositivity or the levels of CMV-reactive antibodies in populations with and without CAD are not informative in PLWH. The data show that cmvIL-10 should be investigated as a mechanism underlying the effects of CMV. Further studies are required to distinguish the effects of full-length and LAcmvIL-10, and to confirm and extend our findings regarding VCAM-1 and IL-1Ra. This would optimally be achieved in a longitudinal cohort large enough to support multivariable analyses.

## Figures and Tables

**Table 1:** Neither detection of cmvIL-10 nor levels of antibodies binding a cmvIL-10 peptide aligned with CAD at any timepoint up to 36 months prior to CAD diagnosis/selection.

	CAD	No CAD	*p*-Value
*At the time of diagnosis/selection (T0)*			
n	52	50	
Positive cmvIL-10 (n)	5	6	0.76
Positive or trace cmvIL-10 (n) ^a^	12	9	0.63
cmvIL-10 antibody (AU × 10^−3^)	5.1 (1.5–34.8)	6.9 (1.7–44.8)	0.13
CMV lysate antibody (AU)	1019 (1–8598)	776 (1–5311)	0.65
*12 months before diagnosis/selection (T-12)*			
n	48	57	
Positive cmvIL-10 (n)	10	7	0.29
Positive or trace cmvIL-10 (n) ^a^	15	11	0.18
cmvIL-10 antibody (AU × 10^−3^)	5.0 (1.7–50.1)	6.9 (1.5–58.0)	0.41
CMV lysate antibody (AU)	805 (1–9230)	783 (1–5073)	0.66
*24 months before diagnosis/selection (T-24)*			
n	42	58	
cmvIL-10 antibody (AU × 10^−3^)	5.4 (2.0–16)	7.5 (0.95–47.2)	0.45
CMV lysate antibody (AU)	1079 (1–6050)	665 (1–5602)	0.41
*36 months before diagnosis/selection (T-36)*			
n	36	51	
cmvIL-10 antibody (AU × 10^−3^)	5.9 (1.6–48.2)	7.1 (1.0–105)	0.18
CMV lysate antibody (AU)	841 (1–6528)	863 (1–6674)	0.82

Plasma samples were screened to detect cmvIL-10 at T0 and T-12 months. Proportions of samples with measurable or trace cmvIL-10 were compared using Fisher’s Exact tests. Antibodies reactive with a cmvIL-10 peptide or a lysate of CMV-infected fibroblasts were determined at all timepoints as arbitrary units (AU) based on standard samples. Data are presented as median (range) and compared using non-parametric Mann–Whitney tests. ^a^ Levels of cmvIL-10 between the highest and lowest blank on their plate are described as “trace”.

**Table 2:** Detection of cmvIL-10 in plasma associates with some plasma inflammatory markers in PLWH who did not develop CAD at the time of selection and 12 months earlier.

	Positive cmvIL-10	No cmvIL-10 Detected	*p* Value
*T0 with CAD*			
n	5	42	
CMV-lysate antibody (AU)	1120 (180–3227)	892 (1–8598)	0.66
cmvIL-10 antibody (AU × 10^−3^)	4.1 (2.9–14.5)	4.9 (1.5–34.8)	0.63
CXCL10 (pg/mL)	65 (19–500)	66 (13–500)	0.58
sCD14 (μg/mL)	2.5 (1.8–2.9)	2.0 (1.4–5.8)	0.16
VCAM-1(μg/mL)	1.5 (0.49–4.3)	1.2 (0.37–4.3)	0.69
IL-1Ra (ng/mL)	0.67 (0.29–0.93)	1.0 (0.24–12.7)	0.20
*T0 no CAD*			
n	6	41	
CMV-lysate antibody (AU)	1120 (1–2425)	766 (1–5311)	0.87
cmvIL-10 antibody (AU × 10^−3^)	6.6 (3.6–44.8)	6.9 (1.7–32)	0.76
CXCL10 (pg/mL)	**136 (29–383)**	**51 (15–192)**	**0.01**
sCD14 (μg/mL)	1.9 (1.64–2.63)	2.0 (1.1–3.7)	0.49
VCAM-1(μg/mL)	1.5 (0.95–2.5)	1.3 (0.65–4.2)	0.59
IL-1Ra (ng/mL)	**0.38 (0.14–0.70)**	**1.05 (0.22–11.2)**	**0.003**
*T-12 months with CAD*			
n	10	33	
CMV-lysate antibody (AU)	*2132 (127–9230)*	*659 (10–6002)*	*0.08*
cmvIL-10 antibody (AU × 10^−3^)	5.0 (2.4–50.2)	5.0 (1.7–35.7)	0.56
CXCL10 (pg/mL)	*70 (27–203)*	*41 (11–500)*	*0.07*
sCD14 (μg/mL)	2.1 (1.6–3.6)	2.2 (1.3–3.6)	0.70
VCAM-1(μg/mL)	*1.5 (1.1–4.2)*	*1.3 (0.43–4.3)*	*0.06*
IL-1Ra (ng/mL)	0.57 (0.19–6.9)	0.86 (0.22–4.9)	0.34
*T-12 months no CAD*			
n	7	41	
CMV-lysate antibody (AU)	533 (10–1892)	946 (10–5073)	0.18
cmvIL-10 antibody (AU × 10^−3^)	9.0 (3.0–46.2)	5.9 (1.5–48.3)	0.13
CXCL10 (pg/mL)	**119 (52–348)**	**49 (14–208)**	**0.01**
sCD14 (μg/mL)	2.36 (1.4–3.1)	2.0 (1.4–3.1)	0.25
VCAM-1(μg/mL)	*2.1 (1.0–4.3)*	*1.3 (0.72–4.3)*	*0.10*
IL-1Ra (ng/mL)	1.3 (0.30–2.0)	0.81 (0.21–5.4)	0.34

Levels of CMV-reactive antibodies and inflammatory/cardiovascular biomarkers were compared between samples deemed positive and negative with respect to cmvIL-10. Samples with trace amounts of cmvIL-10 were omitted. Data are presented as median (range) and analysed using Mann–Whitney non-parametric statistics. *p* values < 0.05 are in bold, with 0.05 < *p* < 0.10 in italics.

**Table 3:** Twelve months before diagnosis/selection, levels of cmvIL-10 peptide antibody correlated directly with sCD14 in PLWH who developed CAD and with IL-1Ra in PLWH without CAD.

	T0		T-12		T-24		T-36	
**CAD**								
	r	*p*	r	*p*	r	*p*	r	*p*
CMV lysate antibody (AU)	**0.36**	**0.01**	*0.28*	*0.06*	0.11	0.49	**0.36**	**0.03**
sCD14 (μg/mL)	0.15	0.28	**0.37**	**0.01**	*0.26*	*0.09*	0.16	0.35
CXCL10 (pg/mL)	−0.08	0.58	0.23	0.11	0.25	0.11	0.18	0.29
VCAM-1 (μg/mL)	0.07	0.62	**0.40**	**0.005**	**0.33**	**0.04**	*0.31*	*0.07*
IL-1Ra (ng/mL)	0.07	0.64	0.08	0.61	0.25	0.12	0.22	0.20
**No CAD**								
CMV lysate antibody (AU)	*0.27*	*0.06*	0.11	0.42	0.03	0.83	0.23	0.11
sCD14 (μg/mL)	0.18	0.22	0.12	0.36	0.19	0.14	−0.08	0.58
CXCL10 (pg/mL)	0.13	0.37	0.13	0.32	0.22	0.10	0.14	0.32
VCAM-1 (μg/mL)	0.13	0.37	0.21	0.11	**0.34**	**0.01**	**0.33**	**0.02**
IL-1Ra (ng/mL)	*0.25*	*0.08*	**0.34**	**0.01**	0.21	0.12	0.19	0.18

Non-parametric Spearman’s rank correlations (r) and associated *p*-values are shown. cmvIL-10 peptide antibody was assessed in arbitrary units (AU). *p* values < 0.05 are in bold, with 0.05 < *p* < 0.10 in italics.

## Data Availability

De-identified data are available on request.

## Supplementary Materials

The following supporting information can be downloaded at: https://www.mdpi.com/article/10.3390/pathogens15020192/s1.
