# Supplementary material for: An Evaluation of IL-10 Encoded by Cytomegalovirus in the Prediction of Coronary Artery Disease in People Living with HIV
Source: Pathogens. 2026 Feb 9;15(2):192. doi: 10.3390/pathogens15020192 (PMC12943061; doi:10.3390/pathogens15020192)
Supplement: Supplementary file 1 [file pathogens-15-00192-s001.zip › pathogens-4096312-supplementary.pdf]

Supplementary Table S1A: GenBank accession numbers for protein sequences used to perform alignments during cmvIL-10 peptide design. These are presented as Figure 1.

**Organism**	**Strain/ Species**	**GenBank Accession No.**
HHV5	CMV AD169	ACL51173.1
HHV5	CMV Towne	ACM48082.1
HHV5	CMV Toledo	AAR31534.1
HHV5	CMV JHC	ADV04421.1
HHV5	CMV Davis	AFR54754.1
HHV5	CMV Merlin	YP_081552.1
HHV4	Epstein-Barr Virus	CAD53385.1
Human	*Homo sapiens*	AAA63207.1
Mouse	*Mus musculus*	AAA39274.1

HHV= Human Herpesvirus CMV = cytomegalovirus

Supplementary Table S1B: In an unbiased Protein BLAST search using default parameters, the closest matches to our peptide were IL-10 encoded by isolates of CMV (HHV5), followed by unrelated proteins from environmental organisms. Other matches generated lower scores.

**Representative sequence**	**optimal match to**	**Total Score**	**E value**	**% ident**	**Accession**
Selected peptide	LQREDDYSVWLDGTVVKGC				
interleukin-10 [HHV5]	LQREDDYSVWLDGTVVKGC	66	6.00E-11	100	AFR55588.1
IL-10F [HHV5]	LQREDDYSVWLDGTVVKGC	66	6.00E-11	100	ABK06387.1
Chain L, IL-10-LIKE PROTEIN [HHV 5]	LQREDDYSVWLDGTVVKGC	66	6.00E-11	100	1LQS_L
latency associated cmvIL-10 [HHV5]	LQREDDYSVWLDGTVVKGC	66	6.00E-11	100	ACR49217.1
IL-10E [HHV 5]	LQREDDYSVWLDG	47.3	2.00E-04	100	ABK06386.1
SOS response-associated peptidase [*Natronogracilivirga saccharolytica*]	REDDYSIWLD	36.3	1.6	90	WP_210513017.1
Golgi mannosyltransferase complex subunit [*Rhizopus stolonifer*]	LEKEDDYVLWLDGDVV	35.8	2.3	68.75	RCH82191.1
Hypothetical protein G6F56_000281 [*Rhizopus delemar*]	LEKEDDYVLWLDGDVV	35.8	2.3	68.75	KAG1474581.1
Hypothetical protein NDN08_006447 [*Rhodosorus marinus*]	DVSVWLDGTVVK	35.4	3.3	91.67	KAJ8902039.1
DUF1559 domain-containing protein [*Caulifigura coniformis]*	REEDYSVWVDGGV	35	4.6	76.92	WP_197453421.1

Supplementary Table S2A: Levels of CMV-reactive antibodies and inflammatory/cardiovascular biomarkers did not align with a diagnosis of CAD made 12 months after sample collection.

	**PLWH with CAD**	**PLWH without CAD**	**p-value**
n	47	52	
Nadir CD4 T-cells/µl	110 (5-618)	132 (1-494)	0.48
Time on ART (years)	9.8 (1.1-27.5)	8.5 (1.3-25)	0.23
Ever received abacavir	29/18	20/32	**0.03**
CMV lysate antibody (AU)	713 (0-9230)	800 (0-5073)	0.92
sCD14 (µg/mL)	2.2 (1.3-3.6)	2 (1.2-3.1)	0.27
LBP (µg/mL)	8.6 (2.8-25)	8 (2.5-21)	0.58
CXCL10 (pg/mL)	45 (11-500)	51 (14.4-364)	0.27
IL-1RA (ng/mL)	0.9 (0.2-6.9)	0.8 (0.3-7.3)	0.89
VCAM-1 (µg/mL)	1.3 (0.4-4.3)	1.3 (0.7-4.3)	0.80
IL-6 (pg/mL)	2.5 (0.9-10)	1.8 (0.4-8.5)	**0.02**

Data are presented as median (range) and analysed used Mann Whitney tests. CMV lysate antibody is presented as arbitrary units (AU). *p*-values<0.05 are in bold.

Supplementary Table S2B: Plasma levels of inflammatory and cardiac biomarkers correlated with CMV-reactive antibodies (in AU) in PLWH with a diagnosis of CAD made 12 months after sample collection.

	PLWH with CAD		PLWH without CAD	
	r	p	r	p
n	47		52	
Nadir CD4 T-cells/µl	**-0.43**	**0.002**	-0.15	0.28
Time on ART (years)	*0.27*	*0.07*	0.13	0.37
sCD14 (µg/mL)	**0.37**	**0.01**	0.04	0.80
LBP (µg/mL)	**0.43**	**0.003**	-0.04	0.79
CXCL10 (pg/mL)	**0.38**	**0.01**	0.05	0.71
IL-6 (pg/mL)	**0.30**	**0.04**	0.04	0.80
VCAM-1 (µg/mL)	0.23	0.13	0.09	0.55
IL-1Ra (ng/mL)	-0.05	0.77	0.08	0.56

Non-parametric Spearman's rank correlations (r) and p values are shown. *p*-values <0.05 are bold with 0.05<*p*<0.1 are in italics.
